# Strangulated bowel obstruction caused by an ileo-ileal knot: a rare case report

**DOI:** 10.1186/s40792-023-01724-6

**Published:** 2023-08-08

**Authors:** Satoko Umetsu, Shigeru Shibata, Harue Akasaka, Shinji Tsutsumi, Chiaki Uchida, Hirokazu Ogasawara

**Affiliations:** 1 Department of Gastroenterological Surgery, Hirosaki General Medical Center, 1-Tomino-Cho, Hirosaki, 036-8545 Japan; 2 Department of Surgery, Kuroishi General Hospital, 1-70-Kitami-Cho, Kuroishi, 036-0541 Japan

**Keywords:** Ileo-ileal knot, Intestinal knot, Strangulated bowel obstruction

## Abstract

**Background:**

Intestinal knot formation is a condition wherein two segments of the intestine are knotted together; however, reports of small-intestinal ileo-ileal knot formation are rare.

**Case presentation:**

The patient was a 62-year-old Asian male with a history of endoscopic colorectal adenoma resection and a spontaneous pneumothorax. The patient had no history of a laparotomy. He consulted his local doctor with the chief complaint of abdominal pain and was admitted to our hospital with suspicion of an acute abdomen. The abdomen had muscular guarding with tenderness and rebound tenderness. Contrast-enhanced computed tomography (CT) showed torsion of the mesentery of the small intestine with poor contrast filling. The patient was referred to our department with strangulated bowel obstruction and underwent an emergency laparotomy. Intraoperative findings revealed that two segments of the ileum were wrapped around each other to form a knot, and the strangulated small bowel was necrotic. After the release of the knot, partial resection of the small intestine was performed from 220 cm distal to the ligament of Treitz to 80 cm proximal to the cecum. The patient had a good postoperative course and was discharged on the 11th postoperative day.

**Conclusion:**

Ileo-ileal knots should be considered as part of the differential diagnosis when treating strangulated bowel obstruction.

## Background

Intestinal knots can cause intestinal obstruction when two intestinal segments wrap around each other to form a knot, resulting in severe ischemia and obstruction. Most case reports have discussed knots that formed between the ileum and sigmoid colon. However, reports of ileo-ileal knots (IIK) are rare [1, 2]. Moreover, the etiology and risks for the development of IIKs remain unclear. In this report, we describe a case of a patient with an IIK that had a favorable outcome following early surgery. Moreover, a literature review on IIKs is described.

## Case presentation

**Fig. 1:**
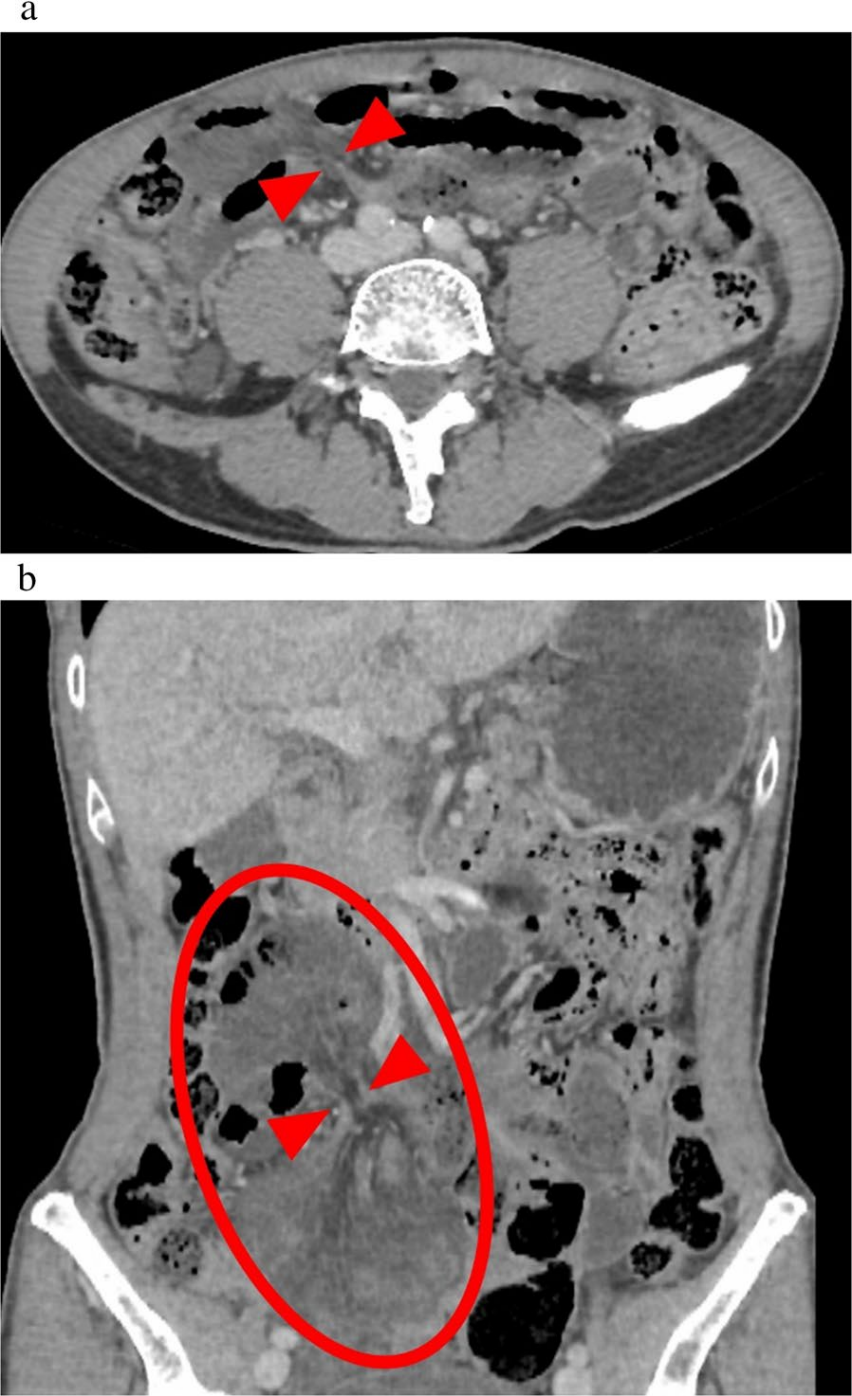
**a** The mesentery of the small intestine is shown twisted (red arrow), with reduced contrast enhancement of the intestinal wall. **b** The small intestine is shown twisted and constricted (red arrow), resulting in decreased contrast enhancement of the intestinal wall in a figure-8 pattern centered at the site (oval)

The patient was a 62-year-old male with a history of endoscopic resection of colorectal adenoma and spontaneous pneumothorax. The patient had no history of a laparotomy. He consulted his local doctor with the chief complaint of abdominal pain and was admitted to our hospital with an acute abdomen. The abdomen had muscular guarding with tenderness and rebound tenderness. His vital signs were normal, including a pulse rate of 73 bpm, blood pressure of 125/73 mmHg, temperature of 37.2 °C, and oxygen saturation of 100% on room air. Blood tests revealed a normal white blood cell count and C-reactive protein level. Contrast-enhanced computed tomography (CT) revealed torsion of the mesentery of the small intestine with poor contrast enhancement (Fig. 1a, b). The coronal section showed an 8-shaped small intestine with reduced contrast enhancement and surrounding ascites (Fig. 1b).

**Fig. 2.**
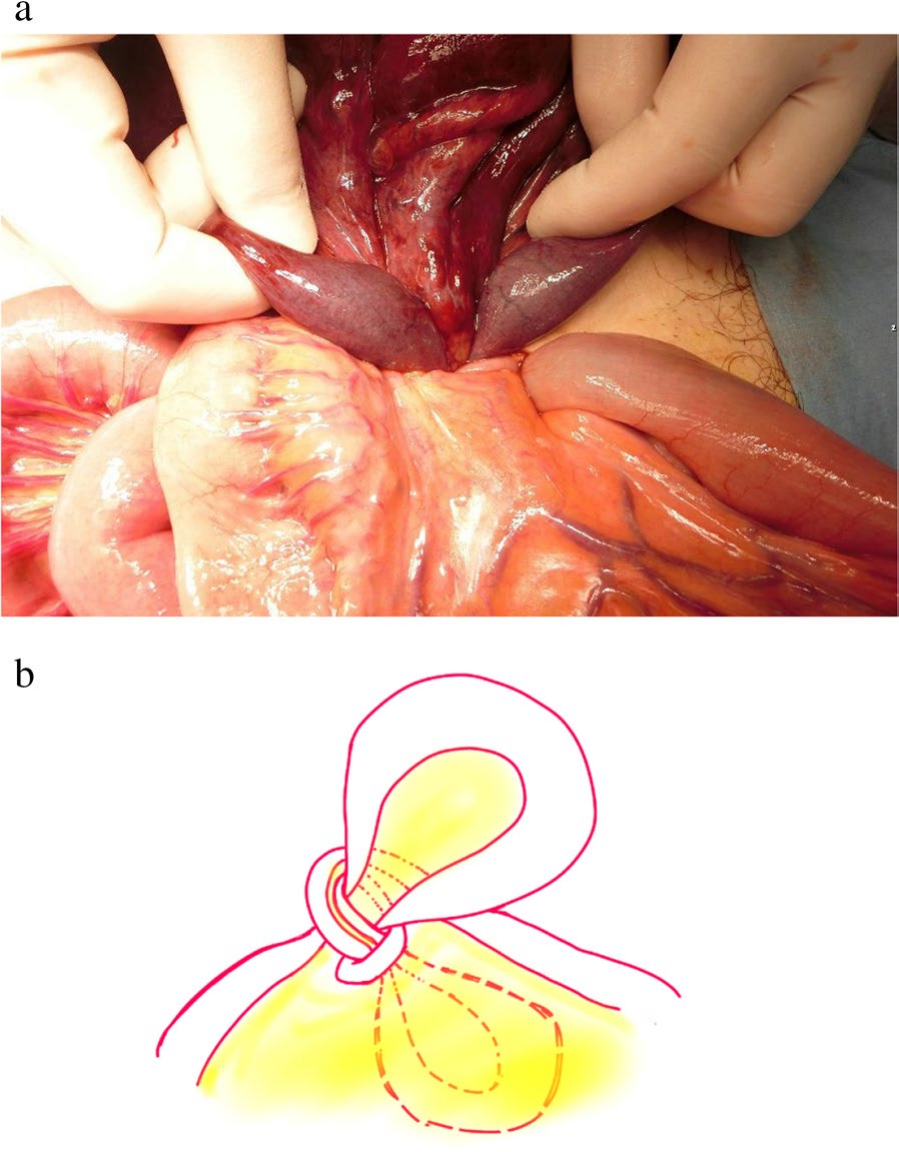
**a** The ileal sections are seen wrapped around each other to form a knot, and the strangulated intestine is visibly necrotic. **b** Schematic representation of the knot

**Fig. 3:**
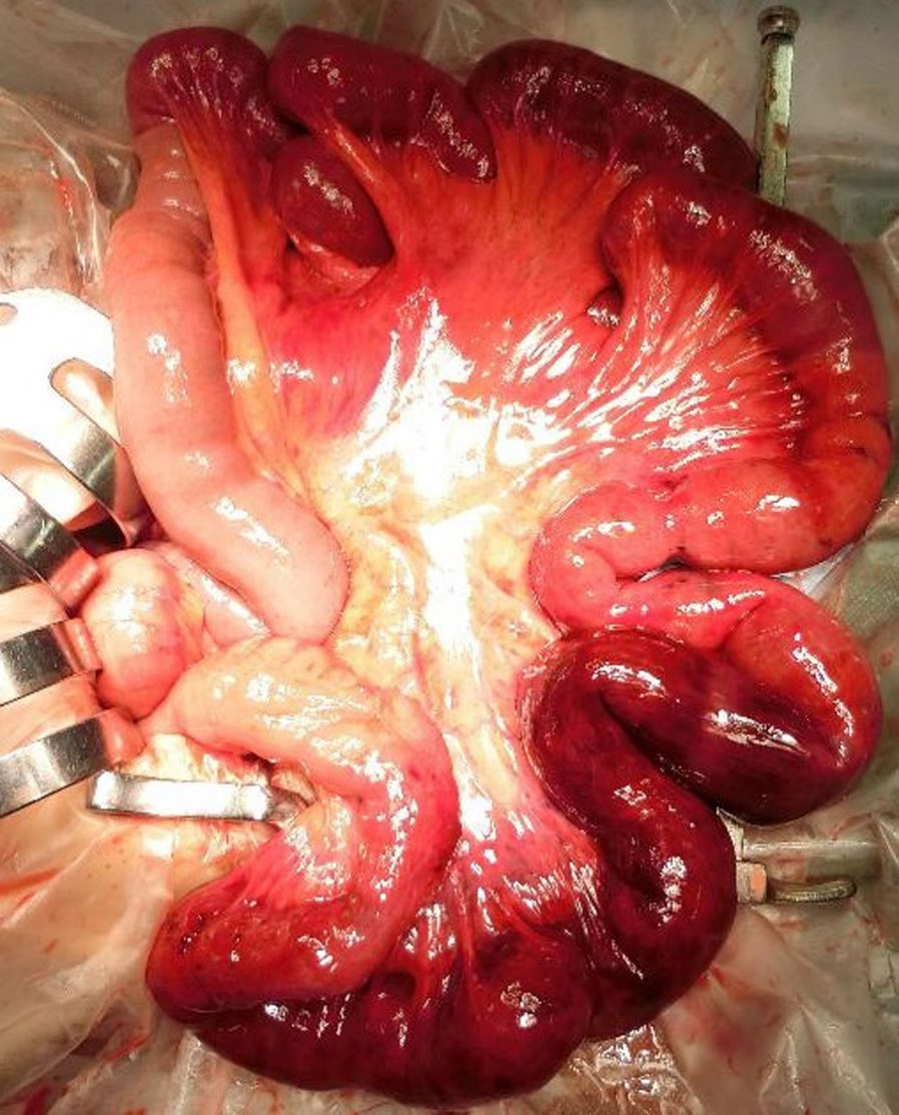
The ileum after the knot was released

The patient was referred to our department with a diagnosis of strangulated bowel obstruction, and an emergency laparotomy was performed within 6 h of symptom onset. Intraoperative findings showed that the ileal loops were wrapped around each other to form a knot, and the strangulated intestine was necrotic (Fig. 2a, b). When the knot was released, two segments of the ileum were visibly necrotic (Fig. 3). Partial resection of the small intestine was performed from 220 cm distal to the ligament of Treitz to 80 cm proximal to the cecum.

Macroscopic specimen findings showed that the resected intestinal tract was approximately 150 cm long, and most of the mucosal surface was black. The histopathological examination revealed congestion and ischemic necrosis.

The postoperative course was uneventful, and the patient was discharged on the 11th postoperative day.

## Discussion

Intestinal knots are obstructions formed by two mobile intestinal loops that wrap around each other, either mutually or by one strangulating the other, and are associated with severe impairment of the perfusion of the intestinal tract. This condition was first mentioned by Riverius in the sixteenth century and reported in more detail by Rokitansky in 1836.

Taylor [3] classified true knots as those in which one of the two intestinal loops passes through the crossing aperture and then wraps around the other, and intertwined (pseudo) knots as those in which the other does not pass through the crossing aperture. True knots generally form between the small intestine and sigmoid colon; however, knots forming between small intestinal loops are rare [3–5]. In the present case, a true knot was formed between two small intestinal loops.

Although the etiology of IIKs is unknown, anatomic factors such as decreased mesenteric fat, elongated mesenteries, and narrow bases [6] and dietary factors such as fasting habits, high fiber, and a single bulky meal have been implicated in the development of ileo-sigmoid knots [5]. It is speculated that the anatomical risk factors increase the mobility of the intestinal tract, and the dietary factors cause a large amount of content to flow into the small intestine in a short period of time, resulting in increased intestinal peristalsis and leading to knot formation [7, 8].

In the present case, although the patient had a normal body mass index (BMI) of 19 kg/m^2^, intraoperative findings revealed an oversized mesentery and low mesenteric fat content, and patient history revealed a habit of consuming large amounts of food in a short period of time. Thus, both anatomic and dietary risk factors were present in this case.

A search of PubMed and Google Scholar databases revealed 13 cases of IIKs with true knot formation that have been reported from 2010 to 2022 [4, 6–16], including this case (Table 1). Most reported cases were from India (six cases) [8, 9, 11–14], followed by Japan (three cases, including this case) [4, 7]. The remaining four were from Greece [6], Ethiopia [10, 16], and Malaysia [15].

**Table 1:** Details of 13 cases of ileo-ileal knot including our case

Author	Nation	Year	Age/sex	Chief compliant	Surgical history	Preoperative diagnosis	Duration until surgery	Knot point	Operation	Untying before resection	Outcome
Uday	India	2012	68/M	Vomiting, distention, constipation, abdominal pain	None	Strangulated bowel obstruction	48 h	15 cm from ileum end	Resection	Yes	Discharged on 8POD
Andromanakos	Greece	2014	26/M	Abdominal pain	None	Strangulated bowel obstruction	6 h	ileum	Resection	Yes	Discharged on 15POD
Abebe	Ethiopia	2015	55/F	Abdominal pain	None	Strangulated bowel obstruction	48 h	8 cm from ileum end	Resection	No	Discharged on 14POD
Gopivallabh	India	2016	54/M	Swelling in the right groin, abdominal pain, vomiting	Appendectomy	Right obstructed inguinal hernia, small bowel obstruction	NA	30 cm from ileum end	Resection	No	Discharged on 6POD
Kalaichelvan	India	2016	65/M	Abdominal pain, nausea, vomiting	None	Strangulated small bowel obstruction	48 h	ileum	Release obstruction	Yes	Discharged on 8POD
Taniguchi	Japan	2017	80/F	Abdominal pain	Colostomy, appendectomy	Strangulated bowel obstruction	6 h	10 cm from ileum end	Resection	Yes	Discharged on 12POD
Prabhakar	India	2019	22/M	Distension, constipation, vomiting, abdominal pain	Left inguinal hernia	Strangulated small bowel obstruction	48 h	30 cm from ileum end	Resection, double-barrel ileostomy	No	Discharged on 6POD
Rajesh	India	2019	23/M	Abdominal pain, vomiting, constipation	Hirschsprung	Strangulated bowel obstruction	48 h	20 cm from ileum end	Release obstruction	-	Discharged on 8POD
Beg	India	2020	55/F	Non-passage of feces and flatus, abdominal pain	None	Intussusception and Strangulated small bowel obstruction	48 h	Ileum*	Resection	No	Discharged on 6POD
Sohali	Malaysia	2020	17/F	Abdominal pain, vomiting	Appendectomy	Strangulated small bowel obstruction	24 h	5 cm from ileum end	Resection	No	Discharged on 5POD
Kanamori	Japan	2021	89/F	Abdominal pain, vomiting	Cesarean section	Strangulated bowel obstruction	7 h	10 cm from ileum end	Resection	No	Discharged on 13POD
Mohammed	Ethiopia	2021	18/F	Abdominal pain	None	Ruptured ovarian cyst	12 h	ileum	Resection	No	Discharged on 6POD
Present case	Japan	2021	62/M	Abdominal pain	None	Strangulated bowel obstruction	8 h	80 cm from ileum end	Resection	Yes	Discharged on 11POD

*A loop of ileum was intussuscepting into the ileal loop and the whole loop within loop intussuscepting into the caecum and ascending colon. An ileo-ileal knot was present in the bowel loop just proximal to intussusception

Of the 13 previously reported cases, 3 were described as having adhesions related to intestinal knots [7, 11, 15]. Others stated that increased mobility of the intestinal tract promoted knot formation [4, 8, 14]. Since the majority of cases of knot formation occurred within 30 cm proximal of the ileocecal area, it was hypothesized that "moderate adhesions and exaggerated intestinal peristalsis" promote knot formation [7]. However, in the present case, the knot was formed 80 cm proximal to the cecum, and there were no adhesions. It is important to note that some IIKs may develop without any associated adhesions. We believe that the most important factor for IIK formation is increased intestinal motility and that adhesions may or may not be relevant.

Preoperative diagnosis of IIKs is very difficult, and in all reported cases, the diagnosis was made intraoperatively. In our patient, the preoperative diagnosis was strangulated bowel obstruction, and the diagnosis of IIK was also only made intraoperatively. Preoperatively, we had no knowledge of IIK and were unable to make the diagnosis. However, retrospectively, the CT scan showed that the small intestine formed a double-closed loop. In countries with limited medical resources, CT scans are not frequently conducted, resulting in few confirmed cases; however, as noted by Kanamori et al., the presence of a double-closed loop may be a specific finding of IIKs [7].

Although most cases were operated within 6 to 48 h after symptom onset, 11 of 13 cases required intestinal resections. The need for intestinal resection in IIK does not seem to correlate with the onset time. As noted in two cases [12, 14], only obstruction release was required even though two days had passed since the onset of symptoms. Therefore, it is speculated that the need for intestinal resection depends not on the time of symptom onset but on the degree of strangulation associated with knot formation. Therefore, if strangulation due to the knot formation is severe, even a short time after onset, intestinal resection becomes inevitable. Given that it is currently impossible to evaluate the level of strangulation using available medical technology, early treatment is crucial.

There is no clear consensus on the appropriate surgical method for IIKs regarding the release of the knot before resection. Some studies recommend en bloc resection without relieving the obstruction because of the risk of necrotic material entering the systemic circulation and intestinal perforation [9, 10, 12, 17]. Conversely, another set of studies suggested releasing the obstruction first to avoid excessive intestinal resection [14, 18]. In two reports, surgery was carried out without intestinal resection, and benefits were noted by releasing the knot first [12, 14]. In this case, the small bowel knot was released to minimize the length of resected. The patient's general health should be considered, and surgery should be performed accordingly to minimize resection of the intestinal tract, with or without knot release.

## Conclusions

Here, we report a case of strangulated bowel obstruction caused by an IIK. This should be considered when treating strangulated bowel obstruction.

## Data Availability

All data generated during this study are included in this published article.

## Abbreviations

BMI: Body mass index
CT: Computed tomography
IKK: Ileo-ileal knot
